# Dynamics of Antibodies to Ebolaviruses in an *Eidolon helvum* Bat Colony in Cameroon

**DOI:** 10.3390/v14030560

**Published:** 2022-03-09

**Authors:** Dowbiss Meta Djomsi, Flaubert Auguste Mba Djonzo, Innocent Ndong Bass, Maëliss Champagne, Audrey Lacroix, Guillaume Thaurignac, Amandine Esteban, Helene De Nys, Mathieu Bourgarel, Jane-Francis Akoachere, Eric Delaporte, Ahidjo Ayouba, Julien Cappelle, Eitel Mpoudi Ngole, Martine Peeters

**Affiliations:** 1 Laboratoire de Virologie-Cremer, Institut de Recherches Médicales et d’Études des Plantes Médicinales (IMPM), Yaoundé P.O. Box 13033, Cameroon; medjodow@yahoo.fr (D.M.D.); mbaflaubertz75@gmail.com (F.A.M.D.); ssabgnodn@yahoo.com (I.N.B.); 2 Transvihmi, Institut de Recherche pour le Développement (IRD), University of Montpellier, Inserm, 34394 Montpellier, France; maeliss.champagne@ird.fr (M.C.); audrey.lacroix@ird.fr (A.L.); guillaume.thaurignac@ird.fr (G.T.); amandine.esteban@ird.fr (A.E.); eric.delaporte@ird.fr (E.D.); ahidjo.ayouba@ird.fr (A.A.); 3 ASTRE, CIRAD, Harare, Zimbabwe; helene.de_nys@cirad.fr (H.D.N.); mathieu.bourgarel@cirad.fr (M.B.); 4 ASTRE, CIRAD, INRAE, University of Montpellier, 34398 Montpellier, France; 5 Department of Microbiology and Parasitology, University of Buea, Buea P.O. Box 63, Cameroon; janetatah@gmail.com (J.-F.A.); julien.cappelle@cirad.fr (J.C.)

**Keywords:** ebola, Africa, bat, *Eidolon helvum*, Cameroon, virus, antibody

## Abstract

The ecology of ebolaviruses is still poorly understood and the role of bats in outbreaks needs to be further clarified. Straw-colored fruit bats (*Eidolon helvum*) are the most common fruit bats in Africa and antibodies to ebolaviruses have been documented in this species. Between December 2018 and November 2019, samples were collected at approximately monthly intervals in roosting and feeding sites from 820 bats from an *Eidolon helvum* colony. Dried blood spots (DBS) were tested for antibodies to Zaire, Sudan, and Bundibugyo ebolaviruses. The proportion of samples reactive with GP antigens increased significantly with age from 0–9/220 (0–4.1%) in juveniles to 26–158/225 (11.6–70.2%) in immature adults and 10–225/372 (2.7–60.5%) in adult bats. Antibody responses were lower in lactating females. Viral RNA was not detected in 456 swab samples collected from 152 juvenile and 214 immature adult bats. Overall, our study shows that antibody levels increase in young bats suggesting that seroconversion to Ebola or related viruses occurs in older juvenile and immature adult bats. Multiple year monitoring would be needed to confirm this trend. Knowledge of the periods of the year with the highest risk of Ebolavirus circulation can guide the implementation of strategies to mitigate spill-over events.

## 1. Introduction

Since the first recognized Ebolavirus disease (EVD) outbreak in 1976 in the Democratic Republic of Congo (DRC), more than 30 outbreaks have been reported in Africa [1,2]. The majority of EVD outbreaks occurred in remote areas and remained geographically restricted, but the recent outbreaks in West Africa (December 2013 to March 2016) and in Eastern DRC (August 2018 to June 2020) clearly showed that thousands of individuals can be infected over large geographic areas [3]. From these larger epidemics, it is evident that certain EVD outbreaks can be linked to individuals who recovered from the disease, even after more than five years [4,5].

Nevertheless, the majority of EVD outbreaks are most likely the result of independent zoonotic transmission events, and bats are considered as a possible reservoir species [6]. Viral RNA and antibodies to Zaire Ebola virus (EBOV) were detected in three frugivorous bat species (*Epomops franqueti*, *Hypsignathus monstrosus,* and *Myonycteris torquatus*) during EVD outbreaks in Gabon and the Republic of Congo [7]. Surveys of bats in West, Central, and East Africa showed the presence of antibodies to ebolaviruses in at least eight frugivorous species and one insectivorous genus (*Mops* sp.) [8,9,10,11]. Despite the absence of direct evidence of exposure to infected bats in the index cases, two EVD outbreaks have been linked to bats; i.e., Luebo (DRC) in 2007 and the major outbreak in West Africa in 2013 [12,13]. Viral RNA from other filoviruses has also been detected in bats; Marburg virus (MARV) in Africa [14,15,16], Bombali virus (BOMV) in insectivorous bats (*Mops condylurus* and *Chaerephon pumilus*) in Africa [17,18,19], Lloviu virus in Europe [20,21], and other filoviruses including Reston Ebolavirus (RESTV) in China and the Philippines [22,23]. MARV has been isolated from Egyptian fruit bats (*Rousettus aegyptiacus*), the assumed reservoir species [14]. Apparently, bats support MARV virus replication with no apparent disease as observed in experimentally infected bats [24,25].

Ebolaviruses are most likely cleared from their hosts and can therefore only be detected for a limited time period, explaining partly the low number of bats in which viral RNA was detected. Despite the increasing number of EVD outbreaks and their increasing importance, the ecology of ebolaviruses and the role of bats in outbreaks needs to be further clarified. Bats represent a source of spillover into human populations or other intermediate animal hosts through hunting or indirect exposure to fruits contaminated by saliva, urine, or feces [7,12,26,27,28]. Straw-colored fruit bats (*Eidolon helvum*) are the most common fruit bats in Africa and form large seasonal aggregations of several thousand (>100,000) individuals. They roost in many forest and savanna zones and can also be found in urban areas in large fruit trees close to human settlements and the colonies can migrate over long distances [29,30]. Humans can thus be exposed to a wide diversity of viruses harbored by *Eidolon helvum* bats. No Ebolavirus RNA has been detected yet, but several studies reported antibodies to ebolaviruses in *Eidolon helvum* bats [11]. Seasonality of viral shedding in bats has been reported for paramyxoviruses, coronaviruses, Marburgvirus and has been suggested for other filoviruses [26,31,32,33]. Given the potential seasonality of viral shedding and rapid viral clearance after infection, longitudinal surveillance of bat populations is thus needed. Here we monitored a colony of *Eidolon helvum* bats in Yaoundé, the capital city of Cameroon, for antibodies to ebolaviruses and viral shedding over a one-year period.

## 2. Materials and Methods

### 2.1. Collection Sites

Samples were collected from free-ranging *Eidolon helvum* bats at approximately monthly intervals (median four weeks, range two to eight weeks), between December 2018 and November 2019, at a roosting site in Yaoundé, the capital city of Cameroon, and at a feeding site at 40 km distance. Bats were captured as previously described using mist nets [11]. Whole blood (volumes ranged between 50–250 μL, depending on the size of bats) was collected as dried blood spots (DBS) by venipuncture from the propatagial or brachial vein, dropped on Whatman 903 filter paper (GE Healthcare, Feasterville-Trevose, PA, USA) and air-dried [11]. Rectal and oral swabs, preserved in RNA-later, were also collected from each bat [11]. For ethical and conservation purposes, bats were released immediately after sampling. Samples were stored in the field at ambient temperature and were subsequently frozen in the laboratory. Data on morphology (measurements of the body, the forearm, the tail, and the metacarpus of the third finger in millimeters, weight, color), sex, age class, and visual species identification were recorded for each bat sampled. Individuals without fully ossified and fused metacarpal-phalangeal epiphyses were classified as juveniles. Males were classified as immature if they lacked enlarged testes and/or distended cauda epididymis, or mature if they were enlarged or distended. The development and morphology of the mammary glands and thoracic (axillary) and pubic nipples were used to classify immature and mature adult females. Pregnancy was assessed by abdominal palpation to determine the presence of a fetus and lactation was confirmed by the extrusion of milk after gentle palpation of the mammary glands and nipples.

Permission to conduct research and to collect samples was obtained from the National Ethics Comitee from Cameroon (N°2018/09/1090/CE/CNERSH/SP).

### 2.2. Molecular Confirmation of Bat Species

The species identification recorded in the field was molecularly confirmed on a subset of bats using DNA extracted from DBS as previously described [11,34]. Briefly, a fragment of approximately 800 base pairs (bp) of the mitochondrial cytochrome b (cytb) was amplified using primers adapted from Irwin and colleagues [34,35]. Amplicons were sequenced using the Sanger technique. Sequences were submitted to NCBI for BLAST analysis to identify the most similar bat species. For sequences with no or low similarity (<97%) hits with species in Genbank, phylogenetic tree analysis with reference sequences was performed using maximum likelihood methods with RAxMLv8 [36] implemented in MegAlignPro version 17.2 (DNASTAR. Madison, WI, USA) in order to determine the genus.

### 2.3. Screening for Ebolavirus Antibodies

Dried blood spots (DBS) were tested with a Luminex-based serological assay adapted for bats as previously described [11,34,37]. The assay included nine recombinant proteins, i.e., glycoprotein (GP), nucleoprotein (NP), or viral protein 40 (VP40) for three Ebolavirus species that circulate in Africa (Zaire (EBOV), Sudan (SUDV), and Bundibugyo (BDBV)). Whole blood from DBS was reconstituted as previously described [11,34,37]. Plasma was reconstituted from one DBS spot in 1 mL of incubation buffer, consisting of phosphate-buffered saline (PBS) containing 0.75 mol/L NaCl, 1%(wt/vol) bovine serum albumin (Sigma Aldrich, St. Quentin Fallavier, France), 5% (vol/vol) fetal bovine serum (Gibco-Invitrogen, Cergy Pontoise, France), and 0.2% (vol/vol) Tween-20 (Sigma-Aldrich, Hohenbrunn, Germany). 100 μL of the sample, adjusted at a final plasma dilution of 1/2000, taking into account the hematocrit, was incubated with 50 μL of magnetic beads coated with recombinant protein (2 μg protein/1.25 × 10^6^ beads) in 96-well flat-bottom chimney plates (Greiner bio one, Frickenhausen, Germany) on a plate shaker at 300 rpm for 16 h at 4 °C in the dark. After washing, 0.1 μg/mL of goat anti-bat biotin–labeled IgG (Euromedex, Souffelweyersheim, France) was added to each well and incubated for 30 min at 300 rpm at room temperature. After washing, 50 μL of 4 μg/mL streptavidin-R-phycoerythrin (Fisher Scientific/Life Technologies, Illkirch, France) was added per well and incubated for 10 min at 300 rpm at room temperature. Reactions were read with BioPlex-200 (BioRad, Marnes-la-Coquette, France) or MagPix (Luminex, Austin, TX, US). At least 100 events were read for each bead set, and results were expressed as median fluorescence intensity (MFI) per 100 beads. Samples that showed positive signals were repeated in order to validate the results.

To calculate the proportion of samples reactive for each antigen we used previously calculated cut-off values. We first used less stringent conditions with the general formula based on the MFI values of negative control samples as described previously, i.e., mean of 145 negative samples plus 4 × SD (standard deviation) [11]. Secondly, we used more stringent conditions and determined consensus cut-offs as the mean of cut-offs obtained using three previously described statistical methods used in the absence of well documented positive controls, i.e., change point analysis method and fitted univariate binomial and exponential distributions at a 0.001 risk for error [11,34]. The cut-offs used were those described by Lacroix and colleagues [34] and were calculated on a total of 8741 bats from Guinea, DRC, and Cameroon, including the *Eidolon helvum* colony reported in this study (Supplementary Table S1) [34]. Analyses were performed with R version 4.0.2 software (https://www.r-project.org/, last accessed on 30 November 2021). A sample was considered reactive with an antigen if the MFI value was above the cut-off.

### 2.4. Nucleic Acid Extraction and PCR Screening for Detection of Filoviruses

RNA was extracted from 250 µL of oral and/or rectal swab samples in RNA-later, using the m2000 RNA extraction kits (Abbott, Rungis, France) or Viral QiaAMP (Qiagen, Courtaboeuf, France). cDNA was prepared using the Reverse Transcription System kit with random primers (Promega, Madison, WI, USA), following the manufacturer’s instructions. PCR screening was performed by a semi-nested PCR targeting a 630 bp fragment of the L gene using degenerated primers that detect a wide diversity of filoviruses, as previously described, i.e., Filo-MOD-FWD: TITTYTCHVTICAAAAICAYTGGG and FiloL.conR: ACCATCATRTTRCTIGGRAAKGCTTT in round 1 and Filo-MOD-FWD: TITTYTCHVTICAAAAICAYTGGG, and Filo-MOD-RVS: GCYTCISMIAIIGTTTGIACATT in round 2 [34] Briefly, cDNA was amplified using the GoTaq Hot Start Master Mix PCR kit (Promega, Madison, WI, USA) as follows for first and second PCR rounds: 10 cycles of 92 °C for 20 s, 50 °C for 30 s with −0.5 °C/cycle and 72 °C for 1 min, 35 cycles of 92 °C for 20 s, 50 °C for 30 s and 72 °C for 1 min. Additionally, samples with bands of the expected size were also tested using a previously described Ebola Zaire specific semi-nested PCR assay targeting a 184 bp fragment on the VP35 gene [38].

### 2.5. Logistic Regression for Seropositivity

Fitting Generalized Linear Mixed Models (GLMM) were developed on Rstudio version 1.4.1106 (function: glmer, package: lme4) to explain the presence or absence of antibodies against Ebolavirus antigens (one GLMM model was run for each antigen included in the serological assay), according to sex and age, and for females impact of the reproductive stage (lactation, gestation) was also analyzed in detail. The time period of sample collection was added as a variable with a random effect to take into account the non-independence of samples collected during the same session. Emmeans and ggeffects functions were also used to obtain estimated marginal means for the response and generate predictions for a model by holding the non-focal variable constant and varying the focal variable. The contrast function was used to obtain comparisons and significance (*p*-value) between the different modalities, *p*-value < 0.05 was considered as significant.

## 3. Results

### 3.1. Eidolon Helvum Population

**Figure 1:**
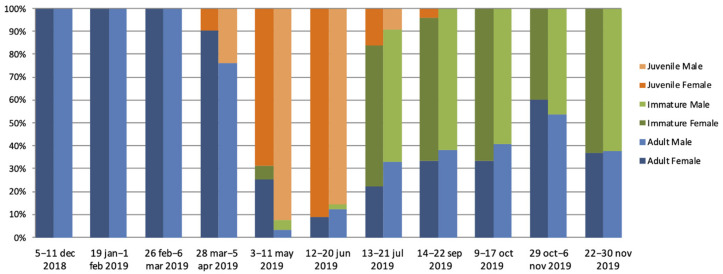
Age distribution per sex for *Eidolon helvum* bats captured at each field mission, shown as percentages. Details on numbers captured at each mission are shown in Supplementary Table S2.

Between December 2018 and November 2019, eleven field missions were conducted to capture *Eidolon helvum* bats, from a bat colony estimated to be of around 100,000 individuals by manual counting at each mission (F. Mba Djonzo, personal observation). Dried blood spots (DBS), rectal and oral swabs were obtained from 820 bats. Overall, 192 (23.4%) and 181 (22.1%) were adult female and male bats, respectively. Adult bats were collected at each session, but between December and March only adults were captured (Figure 1, Supplementary Table S2). Juvenile bats, 108 (13.2%) female and 113 (13.8%) male were mainly captured between March and July and were predominant in capture sessions from May to June. Immature adult bats, 104 (12.6%) females and 122 (14.9%) males, were captured from May to November and were predominant during capture sessions from July to November. Among the adult females, 13/192 (6.8%) were pregnant and they were observed during capture sessions conducted in January, February, and November. Lactation was observed during capture sessions from March to May in a total of 68 (35.4%) adult females. In January, four females were observed with the pup attached.

### 3.2. Antibodies to Ebolaviruses

Dried blood spots from 817 bats were tested for antibodies. Because there were no positive control samples, we used a range to express the number and percentages of reactive samples according to different cutoff calculations (stringent and less stringent) as described in the methods (Supplementary Table S1). Table 1 shows antibody reactivity of all samples to the different Ebolavirus antigens, reactivity was highest with GP proteins, ranging from 5.0% to 8.0% and 16.8% to34.4% for the two Zaire (EBOV-GP-M and EBOV-GP-K) strains, 11.3% to 48.9% for Sudan (SUDV) and 3.9% to 30.9% for Bundibugyo (BDBV). High cross-reactivity was seen among the different GP proteins, the majority of samples reactive with GP-SUDV were also showing reactivity at least one other GP antigen. Reactivities were lower with NPandVP-40 proteins. Only small numbers of samples were simultaneously reactive with two or three different antigens from the same Ebolavirus species.

**Figure 2:**
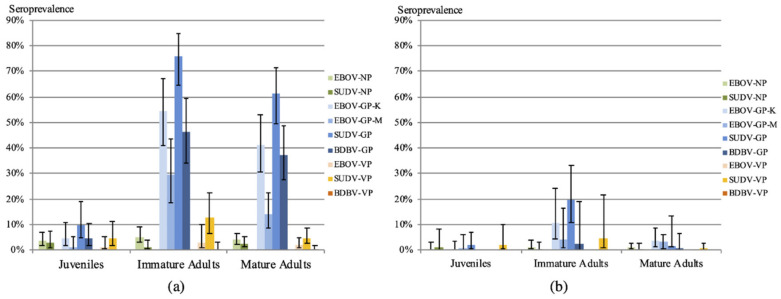
Percentage of bats per age category with antibodies to the different Ebolavirus antigens with less stringent cut-off (4 × standard deviation) (**a**); and stringent cut-off (statistical methods described in methods) (**b**) calculations. Bars correspond to the 95% confidence limits, corresponding *p* values comparing the antibody reactivities for each antigen among the different age categories are shown in Table 3.

Antibody reactivity with the different antigens was analyzed according to age and sex with stringent and less stringent cut-off definitions (Figure 2, Table 2; Supplementary Table S3a,b). In both scenarios, antibodies were absent in almost all juvenile bats. Interestingly, the proportion of samples reactive with GP antigens increased significantly with young age using less-stringent cut-off (*p* < 0.001) and with stringent cut-off for GP-EBOV-K (*p* = 0.0076) and GP-SUDV (*p* = 0.0015) only (Figure 2, Table 3). No differences were observed between females and males for all age categories (Table 3). The proportion of reactive samples with NP and VP proteins was low and differences were only seen for VP-SUDV.

**Figure 3:**
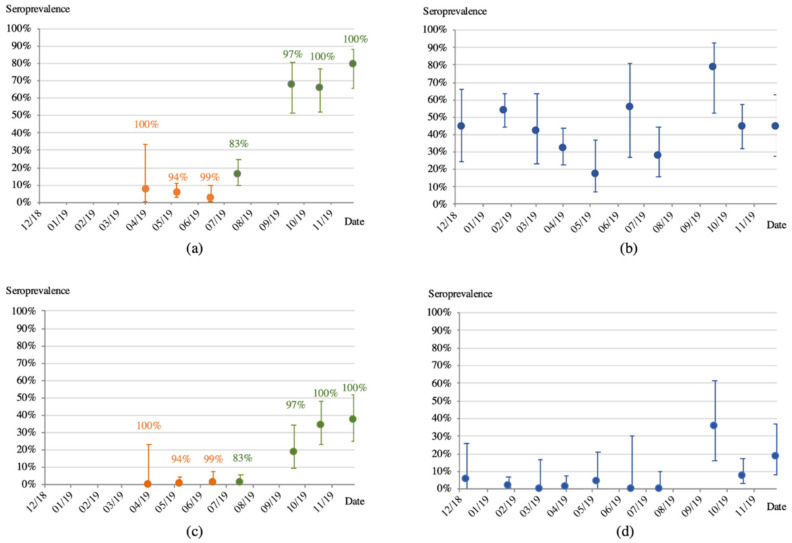
Proportion of samples reactive with the Ebola Zaire Kissidougou glycoprotein (GP-EBOV-k) according to the less stringent cut-off (4 × standard deviation) value for juveniles (orange dots) and immatures (green dots) (**a**); and adults (blue dots) (**b**); and with stringent (statistical methods described in methods) cut-off value for juveniles and immatures (**c**); and adults (**d**). Percentages above the orange and green dots of juvenile and immature bats respectively, correspond to the proportion of the dominant age class during the collection session indicated in the same color of the corresponding dots. Bars correspond to the 95% confidence limits.

Seroprevalence with respect to age at the different time points was analyzed for GP EBOV-K and GP SUDV proteins only because the highest reactivity was seen for these antigens. Figure 3a,c illustrate the proportion of juvenile and immature bats reactive with GP-EBOV-K, and shows that seroconversion occurs at a transition period when juvenile bats are becoming immature adults, i.e., between July and September. Figure 3b,d show that the proportion of adult bats with antibodies did not significantly vary over time. The same trends are observed with both stringent or less stringent cut-offs.

**Figure 4:**
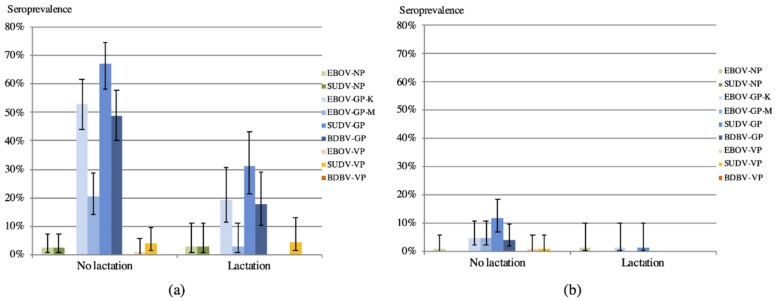
Percentage of adult female bats with antibodies to the different Ebolavirus antigens according to lactation status at capture with less stringent cut-off (4 × standard deviation) (**a**); and stringent cut-off (statistical methods described in methods) (**b**) calculations. Bars correspond to the 95% confidence limits, corresponding *p* values comparing the antibody reactivities for each antigen among lactating and non-lactating females are shown in Table 3.

Variation in antibody response according to the reproductive stage of female adult bats was investigated. The number of bats for which gestation was observed was low (*n* = 13) and could not allow us to draw significant conclusions, but gestation seems not to have an impact on antibody response. Antibody responses were higher in non-lactating bats, and this trend was observed with stringent and less stringent cut-off calculations, although differences were only significant with the less stringent cut-off (*p* ranging between 0.0045 and <0.0001) (Figure 4, Table 3 and Table 4; Supplementary Table S4a,b).

### 3.3. Molecular Screening for the Presence of Ebolaviruses

A total of 456 swab samples from 366 bats (152 juveniles and 214 immature adults) were screened for the presence of viral RNA, because the antibody profiles suggested seroconversion in these age categories. Depending on the availability, oral and rectal swabs were both tested for 87 immature and 3 juvenile bats, only rectal swabs were tested for 147 juvenile and 127 immature bats, for two juvenile bats only oral swabs were tested. Overall, we tested 68.8% (152/221) and 92.5% (214/232) of the juvenile and immature adult bats from the study population for the presence of Ebola viral RNA. None of the 456 swabs tested positive for filovirus viral RNA.

## 4. Discussion

Today, ebolaviruses are a significant public health problem because of the increasing number of outbreaks, the high number of infected individuals, and the wide geographic spread of certain outbreaks in addition to the high mortality rates [3]. Among filoviruses, the role of bats as potential reservoir host species is well established for Marburgvirus (MARV) where a large diversity of MARV viruses has been amplified and sequenced from *Rousettus aegyptiacus* bats [14,15,16,31]. On the other hand, the number of bats from which Ebolavirus RNA was amplified is still restricted to a single study and a handful of samples [7]. However, several studies showed antibodies in at least eight frugivorous bat species and in insectivorous bats from the *Molossidae* family, suggesting that bats play a role in the ecology of ebolaviruses [11]. If ebolaviruses behave like MARV, then the viruses are most likely cleared after infection and the period during which viruses can be detected could thus be very short, explaining partly the low success rate to amplify ebolaviruses in bats [24]. Because sampling over time of the same bat colonies could increase the likelihood to detect viral shedding and identify seasonal trends [39], we conducted a longitudinal surveillance of an *Eidolon helvum* colony, the most common fruit bats in Africa in which antibodies to ebolaviruses have been documented by several studies [11].

Bats are infected with a wide diversity of RNA viruses with pathogenic potential for humans [40] and several studies showed seasonal variations in infection and immunological status according to age, breeding phase, and nutritional stress for henipaviruses, lyssaviruses, and coronaviruses [26,31,41,42,43,44]. The few longitudinal studies on filoviruses showed temporal dynamics in viral shedding for the Marburg virus and in seroprevalence for Marburg and ebolaviruses [39]. For MARV, viral RNA was mainly detected in organs from older juvenile bats and the incidence of MARV infection apparently decreases in the adult population after seven to eight months of age [31]. Serological data on *Rousettus aegyptiacus* in Uganda showed that antibody prevalence to MARV increases with age from 4.1% in young juveniles to 14.8% in older juveniles reaching 21.5% in adult bats [31]. These observations were also confirmed in a colony of *Rousettus aegyptiacus* bats in South-Africa [44]. In the present study, we observed a similar trend in *Eidolon helvum* bats in Cameroon, i.e., antibody prevalence increased with young age, reaching a peak in immature adults. Our results are in line with a study in Madagascar that showed the presence of antibodies to ebolaviruses in *Eidolon dupreanum*, and documented increasing seroprevalence in young bats [45]. The same study showed on age-structured data that seroprevalence decreases late in life [45]. Peaks of filovirus prevalence in juvenile bats were also predicted by [46]. In our study age categories are less detailed and were limited to classification in three major categories (juveniles, immatures, and adults), with no details on the age of adults, and we can thus not provide information on eventual antibody decline in adults over time. Differences in antibody response according to the reproductive cycle in female adult bats have also been reported. Because of the limited number of gestating bats in our study, it was difficult to draw any significant conclusions and compare with data from other studies that observed different antibody prevalence in gestating versus non-gestating bats [26,45]. However, our data clearly show for the first time that antibody prevalence is lower in lactating bats, and this could imply that lower levels of maternal antibodies are transmitted to offspring resulting in higher susceptibility of juveniles to viral infections.

Despite the longitudinal follow up we did not amplify viral RNA in *Eidolon helvum* bats in our study. We only collected rectal and oral swabs and did not euthanize bats to examine the presence of the virus in organs, like the liver or spleen. Nevertheless, viral shedding of MARV has been demonstrated in experimentally infected bats and MARV viral RNA was also amplified from oral swabs in wild bat populations in Sierra Leone [14,25] and in rectal swab samples in South-Africa [47]. Viral loads in swabs are most likely lower than in organs and viruses are thus more difficult to detect. It can thus not be excluded that ebolavirus RNA could be present at some stage in the organs of the *Eidolon helvum* bats.

Our study showed high antibody rates to glycoproteins form three ebolaviruses that circulate in Africa, suggesting that bats encounter at some stage during their life time, probably at end of juvenile and early adult stage, one of the known ebolaviruses or another ebolavirus that cross-reacts with the antigens used. In humans who recovered from Ebolavirus disease in the outbreak in Guinea with the EBOV species, high rates of cross reactivity with other ebolaviruses, SUDV and BDBV, were observed [48]. Cross-reactivity was also observed in a study using convalescent sera from experimentally infected bats but false positive reactivity with other pathogens cannot be excluded [49]. However, many other studies, using different antibody assays also showed the presence of antibodies to ebolaviruses in *Eidolon helvum* bats suggesting that ebolaviruses can circulate in this species [7,10,26,50].

Overall, our study shows that antibody levels increase in young bats with age, suggesting that the highest rates of infection with ebolaviruses occur among older juveniles or young adult bats like for MARV [31]. Multiple year monitoring, and ideally with recapturing of animals and with age determination for adults, would be needed to confirm if this is a trend occurring every year and to increase chances to detect the presence of viral RNA. Our data show also that spill-over events are most likely to occur from young bats and that during the period of the year with the highest presence of older juvenile and immature bats, contact with these bats should be avoided to reduce risks for cross-species transmissions. Although viral transmission via exposure to bat feces on fruits is low, bats are increasingly hunted for consumption, and exposure to infected organs is a more efficient transmission route. Knowing the periods of the year with the highest risk of Ebolavirus circulation can contribute to implementing strategies to reduce spill-over events.

## Figures and Tables

**Table 1:** Numbers and percentages of *Eidolon helvum* bat samples reactive with the different Ebolavirus antigens with stringent (statistical methods) and less-stringent (4 × Standard deviation of negative control samples) cut-offs as described in Methods. The assay used recombinant proteins of Nucleoprotein (NP), Viral Protein-40 (VP40), or Glycoprotein (GP) for different Ebolavirus lineages: Zaire (EBOV), Sudan (SUDV), Bundibugyo (BDBV), and Reston (RESTV). GP proteins from the Mayinga (GP-M) and the Kissidougou (GP-K) strain were used for EBOV.

Antigen		Stringent Cut-Off		Less Stringent Cut-Off	
		N Tested = 817		N Tested = 817	
		n pos	(%)	n pos	(%)
EBOV	NP	8	(0.97)	35	(4.3)
EBOV	GP-K	66	(8.0)	282	(34.4)
EBOV	GP-M	41	(5.0)	138	(16.8)
EBOV	VP	3	(0.4)	22	(2.7)
EBOV	GP + NP	2	(0.3)	13	(1.6)
EBOV	GP + VP	0	(0.0)	8	(1.0)
EBOV	NP + GP + VP	0	(0.0)	4	(0.5)
SUDV	NP	7	(0.9)	20	(2.4)
SUDV	GP	97	(11.3)	401	(48.9)
SUDV	VP	24	(2.9)	60	(7.3)
SUDV	GP + NP	1	(0.1)	6	(0.7)
SUDV	GP + VP	8	(1.0)	46	(5.6)
SUDV	NP + GP + VP	1	(0.1)	2	(0.3)
BDBV	GP	32	(3.9)	254	(30.9)
BDBV	VP	0	(0.0)	2	(0.3)
BDBV	GP + VP	0	(0.0)	1	(0.1)

**Table 2:** Proportion of samples reactive with the different Ebolavirus antigens according to sex and age of the *Eidolon helvum* bats with stringent (statistical methods, see methods) and less- stringent (4 × SD of negative samples) cut-off values. The assay used recombinant proteins of Nucleoprotein (NP), Viral Protein-40 (VP40) or Glycoprotein (GP) for different Ebolavirus lineages: Zaire (EBOV), Sudan (SUDV), Bundibugyo (BDBV) and Reston (RESTV). GP proteins from the Mayinga (GP-M) and the Kissidougou (GP-K) strain were used for EBOV.

Antigen	Juvenile Females		Juvenile Males		Immature Females		Immature Males		Adult Females		Adult Males	
	N = 108		N = 112		N = 103		N = 122		N = 191		N = 181	
	n pos	(%)	n pos	(%)	n pos	(%)	n pos	(%)	n pos	(%)	n pos	(%)
EBOV-NP	1–5	(2.0–4.6)	0–3	(0.0–2.4)	2–4	(1.9–3.9)	1–8	(0.8–6.5)	2–5	(1.0–2.6)	2–10	(1.1–5.5)
SUDV-NP	2–3	(1.8–2.8)	2–4	(1.8–3.6)	0–0	(0.0–0.0)	1–3	(0.8–2.5)	0–5	(0.0–2.6)	2–5	(1.1–2.7)
EBOV-GP-K	1–5	(2.0–4.6)	0–2	(0.0–1.8)	22–56	(21.2–54.3)	24–60	(19.7–49.2)	7–78	(3.6–40.8)	12–81	(6.6–44.8)
EBOV-GP-M	0–1	(0.0–0.9)	1–1	(0.9–0.9)	17–38	(16.3–36.9)	12–37	(9.8–30.3)	6–27	(3.1–14.1)	5–34	(2.8–18.8)
SUDV-GP	3–9	(2.8–8.3)	0–9	(0.0–8.0)	24–74	(23.1–71.8)	35–84	(28.7–68.9)	15–103	(10.9–53.9)	17–122	(9.4–67.4)
BDBV-GP	0–4	(0.0–3.7)	0–3	(0.0–2.4)	14–50	(13.5–48.5)	13–54	(10.6–44.3)	5–71	(2.6–35.7)	5–72	(2.8–39.8)
EBOV-VP	0–3	(0.0–2.8)	0–0	(0.0–0.0)	2–6	(1.9–5.8)	0–5	(0.0–4.1)	1–1	(0.5–0.5)	0–7	(0.0–3.8)
SUDV-VP	2–4	(1.8–3.7)	1–3	(0.9–2.4)	10–16	(9.6–15.5)	8–19	(6.6–15.6)	1–8	(0.5–4.2)	2–10	(1.1–5.5)
BDBV-VP	0–0	(0.0–0.0)	0–0	(0.0–0.0)	0–1	(0.0–0.97)	0–0	(0.0–0.0)	0–0	(0.0–0.0)	0–1	(0.0–0.5)

**Table 3:** *p*-values calculated as described in methods to compare differences in antibody positivity according to age, sex and lactation for females with stringent (statistical methods. see methods) and less- stringent (4 × SD of negative samples) cut-off values. The assay used recombinant proteins of Nucleoprotein (NP). Viral Protein-40 (VP40) or Glycoprotein (GP) for different Ebolavirus lineages: Zaire (EBOV). Sudan (SUDV). Bundibugyo (BDBV) and Reston (RESTV). GP proteins from the Mayinga (GP-M) and the Kissidougou (GP-K) strain were used for EBOV. *p*-values < 0.05 are highlighted in bold.

		NP	NP	GP	GP	GP	GP	VP	VP	VP
		EBOV	SUDV	EBOV- K	EBOV-M	SUDV	BDBV	EBOV	SUDV	BDBV
Juveniles vs. Immature Adults										
	Less stringent CO	0.6825	0.4457	**<0.0001**	**<0.0001**	**<0.0001**	**<0.0001**	0.6247	0.1588	1.0000
	Stringent CO	0.6070	0.4751	**0.0076**	0.2539	**0.0015**	0.9882	1.0000	0.8124	1.0000
Juveniles vs. Mature Adults										
	Less stringent CO	0.9580	0.9641	**<0.0001**	**0.0022**	**<0.0001**	**<0.0001**	0.8820	0.9966	1.0000
	Stringent CO	0.7190	0.5508	0.1154	0.6942	0.1217	0.9897	1.0000	0.4942	1.0000
Immatures vs. Mature Adults										
	Less stringent CO	0.7837	0.4929	**0.0487**	**0.0007**	**0.0066**	0.2218	0.7007	**0.0069**	0.9344
	Stringent CO	0.9496	0.9224	**0.0021**	**0.0382**	**0.0004**	**0.0280**	0.5391	**0.0103**	1.0000
Females vs. Males										
	Less stringent CO	0.2811	0.3938	0.2028	0.5899	0.4111	0.2235	0.9547	0.8093	0.9672
	Stringent CO	0.4464	0.2838	0.8070	0.1041	0.9348	0.1492	0.9987	0.3327	1.0000
No lactation vs. Lactation										
	Less stringent CO	0.8367	0.8367	**<0.0001**	**0.0045**	**<0.0001**	**0.0001**	0.9963	0.9106	1.0000
	Stringent CO	0.6743	1.0000	0.2571	0.9935	**0.0394**	0.9936	0.9963	0.9963	1.0000

**Table 4:** Proportion and percentages of samples reactive with the different Ebolavirus antigens according to reproductive stage of adult female *Eidolon helvum* bats with stringent (statistical methods, see methods) and less- stringent (4 × SD of negative samples) cut-off values. The assay used recombinant proteins of Nucleoprotein (NP), Viral Protein-40 (VP40) or Glycoprotein (GP) for different Ebolavirus lineages: Zaire (EBOV), Sudan (SUDV), Bundibugyo (BDBV) and Reston (RESTV). GP proteins from the Mayinga (GP-M) and the Kissidougou (GP-K) strain were used for EBOV.

Antigen	Gestation		No-Gestation		Lactation		No-Lactation	
	N = 13		N = 178		N = 68		N = 123	
	n pos	(%)	n pos	(%)	n pos	(%)	n pos	(%)
EBOV-NP	0–0	(0.0–0.0)	2–5	(1.1–2.8)	1–2	(1.5–2.9)	1–3	(0.8–2.4)
SUDV-NP	0–0	(0.0–0.0)	0–5	(0.0–2.8)	0–2	(0.02.9)	0–3	(0.0–2.4)
EBOV-GP-K	1–6	(7.7–46.2)	6–72	(3.4–40.4)	1–13	(1.5–19.1)	6–65	(4.9–52.8)
EBOV-GP-M	1–3	(7.7–23.1)	5–24	(2.8–13.5)	0–2	(0.0–2.9)	6–25	(4.9–20.3)
SUDV-GP	1–6	(7.7–46.2)	14–97	(7.9–54.5)	1–21	(1.5–30.9)	14–82	(11.4–66.7)
BDBV-GP	1–5	(7.7–38.5)	4–66	(2.2–37.1)	0–12	(0.0–22.1)	5–59	(4.1–47.9)
EBOV-VP	0–0	(0.0–0.0)	1–1	(0.6–0.6)	0–0	(0.0–0.0)	1–1	(0.8–0.8)
SUDV-VP	0–0	(0.0–0.0)	1–8	(0.6–4.5)	0–3	(0.0–4.4)	1–5	(0.8–4.1)
BDBV-VP	0–0	(0.0–0.0)	0–0	(0.0–0.0)	0–0	(0.0–0.0)	0–0	(0.0–0.0)

## Data Availability

Data are available upon request and data on bat sampling are available on the EBO-SURSY website (https://rr-africa.oie.int/fr/projets/ebo-sursy-fr/, accessed on 6 February 2022).

## Supplementary Materials

The following supporting information can be downloaded at: https://www.mdpi.com/article/10.3390/v14030560/s1. Table S1: MFI cut-off values calculated with different methods as described in Methods section for the different Ebolavirus strains [Zaire (EBOV). Sudan (SUDV). Bundibugyo (BDBV) and Reston (RESTV)] and the different antigens used per virus, Table S2: Distribution of *Eidolon helvum* bats per capture session with respect to sex and age, Table S3a: Numbers, percentages and 95% confidence intervals of *Eidolon helvum* bat samples reactive with the different Ebolavirus antigens according to sex and age and less-stringent cut-off (4 × Standard deviation of negative control samples), Table S3b: Numbers, percentages and 95% confidence intervals of *Eidolon helvum* bat samples reactive with the different Ebolavirus antigens according to sex and age with stringent (statistical methods) cut-offs, Table S4a: Proportion, percentage and 95% confidence intervals of samples reactive with the different Ebolavirus antigens according to reproductive stage of adult female *Eidolon helvum* bats with less- stringent (4 × SD of negative samples) cut-off values and Table S4b: Proportion, percentage and 95% confidence intervals of samples reactive with the different Ebolavirus antigens according to reproductive stage of female adult *Eidolon helvum* bats with stringent (statistical methods, see methods) cut-off values.
