# Supplementary material for: Dynamics of Antibodies to Ebolaviruses in an Eidolon helvum Bat Colony in Cameroon
Source: Viruses. 2022 Mar 9;14(3):560. doi: 10.3390/v14030560 (PMC8951055; doi:10.3390/v14030560)
Supplement: Supplementary file 1 [file viruses-14-00560-s001.zip › viruses-1611660-supplementary.pdf]

SUPPLEMENTARY TABLES

Supplementary table S1. MFI cut-off values calculated with different methods as described in Methods section for the different Ebolavirus strains [Zaire (EBOV). Sudan (SUDV). Bundibugyo (BDBV) and Reston (RESTV)] and the different antigens used per virus. The assay used recombinant proteins of Nucleoprotein (NP). Viral Protein-40 (VP40) or Glycoprotein (GP) for different EBV lineages: Zaire (EBOV). Sudan (SUDV). Bundibugyo (BDBV) and Reston (RESTV). GP proteins from the Mayinga (GP-M) and the Kissidougou (GP-K) strain were used for EBOV. *Mean of negatives was calculated on negative control samples (n=145) that were collected from captive-born insectivorous bats (Carollia perspicillata. n=103) hosted at the Zoo of Montpellier. France. and from two frugivorous species (Pteropus giganteus. n=19; Rousettus aegyptiacus. n=23) from the Zoo of Stuttgart, Germany, as described in De Nys et al, 2018. **The other cut-offs were calculated on a sample set of wild captured bats from Guinee, Cameroon and DRC consisting of DBS samples from 8741 bats as described in Lacroix et al. 2021.

**Antigen**	**Mean+4SD***	**mean of the cutoffs****
NP-EBOV	71	196
GP-EBOV-K	128	609
GP- EBOV-M	307	707
VP40-EBOV	75	177
NP-SUDV	131	258
GP-SUDV	251	1896
VP40-SUDV	88	182
GP-BDBV	99	432
VP40-BDBV	363	664
GP-RESTV	249	140

Supplementary Table S2: Distribution of *Eidolon helvum* bats per capture session with respect to sex and age.

Date of sample collection	Adult females		Immature females		Juvenile females		Adult males		Immature males		Juvenile males		Total
	**n**	**(%)**	**n**	**(%)**	**n**	**(%)**	**n**	**(%)**	**n**	**(%)**	**n**	**(%)**	**N**
5- 11/12 2018	10	(50.0)	0	(0)	0	(0)	10	(50.0)	0	(0)	0	(0)	20
19/1 - 1/2 2019	42	(42.8)	0	(0)	0	(0)	56	(57.1)	0	(0)	0	(0)	98
26/2 - 6/3 2019	8	(47.1)	0	(0)	0	(0)	9	(52.9)	0	(0)	0	(0)	17
28/3 - 5/4 2019	56	(64.3)	0	(0)	6	(6.9)	19	(21.8)	0	(0)	6	(6.9)	87
3- 11/5 2019	21	(14.1)	5	(4.1)	57	(38.5)	2	(1.3)	3	(2.0)	60	(40.5)	148
12 - 20/6 2019	3	(3.6)	0	(0)	31	(37.8)	6	(7.3)	1	(1.2)	41	(50.0)	82
13 - 21/7 2019	15	(11.4)	42	(31.8)	11	(8.3)	21	(15.9)	37	(28.0)	6	(4.5)	132
14 - 22/9 2019	8	(12.1)	15	(22.7)	1	(1.5)	16	(24.2)	26	(39.4)	0	(0)	66
9 - 17/10 2019	5	(10.6)	10	(21.3)	0	(0)	13	(27.7)	19	(40.4)	0	(0)	47
29/10 - 6/11 2019	12	(25.0)	8	(16.7)	0	(0)	15	(31.2)	13	(27.1)	0	(0)	48
22 - 30/11 2019	14	(18.7)	24	(32.0)	0	(0)	14	(18.7)	23	(30.7)	0	(0)	75
Total	194	(23.6)	104	(12.6)	106	(12.9)	181	(22.1)	122	(14.9)	113	(13.8)	820

Supplementary table S3a. Numbers, percentages and 95% confidence intervals of *Eidolon helvum* bat samples reactive with the different Ebolavirus antigens according to sex and age and less-stringent cut-off (4 X Standard deviation of negative control samples).

Antigen	Adult females (n=191)		Adult males (n=181)		Juvenile females (n=108)		Juvenile males (n=112)		Immature females (n=103)		Immature males (n=122)	
	**n+**	**(% ; CI 95%)**	**n+**	**(% ; CI 95%)**	**n+**	**(% ; CI 95%)**	**n+**	**(% ; CI 95%)**	**n+**	**(% ; CI 95%)**	**n+**	**(% ; CI 95%)**
EBOV-NP	5	(2.6; 1.1-5.9)	10	(5.5; 3.0-9.9)	5	(4.63; 1.9-10.4)	3	(2.7; 0.9-7.6)	4	(3.9; 1.5-9.6)	8	(6.6; 3.4-12.4)
SUDV-NP	5	(2.6; 1.1-5.9)	5	(2.8; 1.2-6.3)	3	(2.8; 0.9-7.7)	4	(3.6; 1.40-8.8)	0	(0.0-0.0)	3	(2.5; 0.8-6.9)
EBOV-GP-K	78	(40.8; 34.1-47.9)	81	(44.8; 37.7-52.0)	5	(4.6; 1.9-10.4)	2	(1.8; 0.5-6.3)	56	(54.4; 44.8-63.7)	60	(49.2; 40.5-57.9)
EBOV-GP-M	27	(14.1; 9.9-19.8)	34	(18.8;13.8-25.1)	1	(0.9; 0.2-5.1)	1	(0.9; 0.2-4.9)	38	(36.9; 28.2-46.5)	37	(30.3; 22.9-38.9)
SUDV-GP	103	(53.9; 46.9-60.9)	122	(67.4; 60.3-73.8)	9	(8.3; 4.5-15.1)	9	(8.0; 4.3-14.6)	74	(71.8; 62.5-79.6)	84	(68.9; 60.2-76.4)
BDBV-GP	71	(37.2; 30.6-44.2)	72	(39.8; 32.9-47.1)	4	(3.70; 1.5-9.1)	3	(2.7; 0.9-7.6)	50	(48.5; 39.1-58.1)	54	(44.3; 35.8-53.1)
EBOV-VP	1	(0.5; 0.1-2.9)	7	(3.9; 1.9-7.8)	3	(2.78; 0.9-7.9)	0	(0.0; 0.0-3.3)	6	(5.8; 2.7-12.1)	5	(4.1; 1.8-9.2)
SUDV-VP	8	(4.2; 2.1-8.1)	10	(5.5; 3.0- 9.89)	4	(3.7; 1.5-9.1)	3	(2.7; 0.9-7.6)	16	(15.5; 9.8-23.8)	19	(15.5; 10.2-23.1)
BDBV-VP	0	(0.0 ; 0.0-0.0)	1	(0.6 ; 0.10-3.1)	0	(0.0 ; 0.0-0.0)	0	(0.0 ; 0.0-0.0)	1	(0.9 ; 1.7-5.3)	0	(0.0 ; 0.0-0.0)

Supplementary table S3b. Numbers, percentages and 95% confidence intervals of *Eidolon helvum* bat samples reactive with the different Ebolavirus antigens according to sex and age with stringent (statistical methods) cut-offs.

Antigen	Adult females (n=191)		Adult males (n=181)		Juvenile females (n=108)		Juvenile males (n=112)		Immature females (n=103)		Immature males (n=122)	
	**n+**	**(% ; CI 95%)**	**n+**	**(% ; CI 95%)**	**n+**	**(% ; CI 95%)**	**n+**	**(% ; CI 95%)**	**n+**	**(% ; CI 95%)**	**n+**	**(% ; CI 95%)**
EBOV-NP	2	(1.1; 0.3-3.7)	2	(1.1; 0.30-3.9)	1	(0.9; 0.2-5.1)	0	(0.0; 0.0-0.0)	2	(1.9; 0.5-6.8)	1	(0.8; 0.1-4.5)
SUDV-NP	0	(0.0; 0.0-0.0)	2	(1.1; 0.30-3.9)	2	(1.9; 0.5-6.5)	2	(1.8; 0.5-6.3)	0	(0.0; 0.0-0.0)	1	(0.8; 0.1-4.5)
EBOV-GP-K	7	(3.7; 1.8-7.4)	12	(6.6; 3.83-11.2)	1	(0.9; 0.2-5.1)	0	(0.0; 0.0-0.0)	22	(21.4; 14.6-30.2)	24	(19.7; 13.6-27.6)
EBOV-GP-M	6	(3.1; 1.5-6.7)	5	(2.8; 1.2-6.3)	0	(0.0; 0.0-0.0)	1	(0.9; 0.2-4.9)	17	(16.5; 10.6-24.9)	12	(9.8; 5.7-16.4)
SUDV-GP	15	(7.9; 4.8-12.6)	17	(9.4; 5.9-14.5)	3	(2.8; 0.9-7.9)	0	(0.0; 0.0-0.0)	24	(23.3; 16.2-32.3)	35	(28.7; 21.4-37.3)
BDBV-GP	5	(2.6; 1.1-5.9)	5	(2.8; 1.2-6.3)	0	(0.0; 0.0-0.0)	0	(0.0; 0.0-0.0)	14	(13.6; 8.3-21.5)	13	(10.7; 6.3-17.4)
EBOV-VP	1	(0.5; 0.1-2.9)	0	(0.0; 0.0-2.1)	0	(0.0; 0.0-0.0)	0	(0.0; 0.0-0.0)	2	(1.9; 0.5-6.8)	0	(0.0; 0.0-0.0)
SUDV-VP	1	(0.5; 0.1-2.9)	2	(1.1; 0.3-3.9)	2	(1.9; 0.5-6.5)	1	(0.9; 0.2-4.9)	10	(9.7; 5.4-16.9)	8	(6.6; 3.4-12.4)
BDBV-VP	0	(0.0; 0.0-0.0)	0	(0.0; 0.0-2.1)	0	(0.0; 0.0-0.0)	0	(0.0; 0.0-0.0)	0	(0.0; 0.0-0.0)	0	(0.0; 0.0-0.0)

Supplementary table S4a. Proportion, percentage and 95% confidence intervals of samples reactive with the different Ebolavirus antigens according to reproductive stage of adult female *Eidolon helvum* bats with less- stringent (4 X SD of negative samples) cut-off values.

Antigen	Gestation (n=13)		No gestation (n=178)		Lactation (n=68)		No lactation (n=123)	
	**n+**	**(%; 95%CI)**	**n+**	**(% ; CI 95%)**	**n+**	**(% ; CI 95%)**	**n+**	**(% ; CI 95%)**
EBOV-NP	0	(0.0; 0.0-0.0)	5	(2.81; 1.21-6.41)	2	(2.94; 0.81-10.10)	3	(2.44; 0.83-6.93)
SUDV-NP	0	(0.0; 0.0-0.0)	5	(2.81; 1.21-6.41)	2	(2.94; 0.81-10.10)	3	(2.44; 0.83-6.93)
EBOV-GP-K	6	(46.15; 23.21-70.86)	72	(40.45; 33.51-47.79)	13	(19.12; 11.53-30.01)	65	(52.85; 44.07-61.45)
EBOV-GP-M	3	(23.08; 8.18-50.26)	24	(13.48; 9.23-19.28)	2	(2.94; 0.81-10.10)	25	(20.33; 14.16-28.28)
SUDV-GP	6	(46.15; 23.21-70.86)	97	(54.49; 47.16-61.64)	21	(30.88; 21;17-42;64)	82	(66.67; 57.94-74.38)
BDBV-GP	5	(38.46; 17.71-64.48)	66	(37.08; 30.33-44.38)	12	(17.65; 10;39-28;36)	59	(47.97; 39.33-56.72)
EBOV-VP	0	(0.0; 0.0-0.0)	1	(0.56; 0.10-3.11)	0	(0.0; 0.0-0.0)	1	(0.81; 0.14-4.46)
SUDV-VP	0	(0.0; 0.0-0.0)	8	(4.49; 2.29-8.62)	3	(4.41; 1.51-12.19)	5	(4.07; 1.75-9.16)
BDBV-VP	0	(0.0; 0.0-0.0)	0	(0.0; 0.0-0.0)	0	(0.0; 0.0-0.0)	0	(0.0; 0.0-0.0)

Supplementary table S4b. Proportion, percentage and 95% confidence intervals of samples reactive with the different Ebolavirus antigens according to reproductive stage of female adult *Eidolon helvum* bats with stringent (statistical methods, see methods) cut-off values**.**

**Antigen**	**Gestation (n=13)**		**No gestation (n=178)**		**Lactation (n=68)**		**No lactation (n=123)**	
	**n+**	**(%; 95%CI)**	**n+**	**(% ; CI 95%)**	**n+**	**(% ; CI 95%)**	**n+**	**(% ; CI 95%)**
EBOV-NP	0	(0.0; 0.0-0.0)	2	(1.1; 0.3-4.0)	1	(1.5; 0.3-7.9)	1	(0.8;0.1-4.5)
SUDV-NP	0	(0.0; 0.0-0.0)	0	(0.0; 0.0-0.0)	0	(0.0; 0.0-0.0)	0	(0.0; 0.0-0.0)
EBOV-GP-K	1	(7.7; 1.4-33.3)	6	(3.4; 1.6-7.2)	1	(1.5; 0.3-7.9)	6	(4.9; 2.3-10.2)
EBOV-GP-M	1	(7.7; 1.4-33.3)	5	(2.8; 1.2-6.4)	0	(0.0; 0.0-0.0)	6	(4.9; 2.3-10.2)
SUDV-GP	1	(7.7; 1.4-33.3)	14	(7.9; 4.7-12.8)	1	(1.5; 0.3-7.9)	14	(11.4; 6.9-18.2)
BDBV-GP	1	(7.7; 1.4-33.3)	4	(2.3; 0.9-5.6)	0	(0.0; 0.0-0.0)	5	(4.1; 1.8-9.2)
EBOV-VP	0	(0.0; 0.0-0.0)	1	(0.6; 0.1-3.1)	0	(0.0; 0.0-0.0)	1	(0.8; 0.1-4.5)
SUDV-VP	0	(0.0; 0.0-0.0)	1	(0.6; 0.1-3.1)	0	(0.0; 0.0-0.0)	1	(0.8; 0.1-4.5)
BDBV-VP	0	(0.0; 0.0-0.0)	0	(0.0; 0.0-0.0)	0	(0.0; 0.0-0.0)	0	(0.0; 0.0-0.0)
